# Self-Healing Alginate Hydrogel Formed by Dynamic Benzoxaborolate Chemistry Protects Retinal Pigment Epithelium Cells against Oxidative Damage

**DOI:** 10.3390/gels9010024

**Published:** 2022-12-29

**Authors:** Minhua Liu, Yate Huang, Chunwen Tao, Weijia Yang, Junrong Chen, Li Zhu, Tonghe Pan, Ravin Narain, Kaihui Nan, Yangjun Chen

**Affiliations:** 1 State Key Laboratory of Ophthalmology, Optometry and Vision Science, School of Ophthalmology & Optometry, Wenzhou Medical University, Wenzhou 325027, China; 2 Department of Chemical and Materials Engineering, University of Alberta, Edmonton, AB T6G 2G6, Canada; 3 National Engineering Research Center of Ophthalmology and Optometry, School of Biomedical Engineering, Wenzhou Medical University, Wenzhou 325027, China; 4 National Clinical Research Center for Ocular Diseases, Affiliated Eye Hospital of Wenzhou Medical University, Wenzhou 325027, China

**Keywords:** alginate, self-healing hydrogel, dynamic crosslinking, oxidative stress, retinal pigment epithelium

## Abstract

Oxidative stress is considered as a major factor causing retinal pigment epithelium (RPE) dysfunction and finally leading to retinal diseases such as age-related macular degeneration (AMD). Developing hydrogels for RPE cell delivery, especially those with antioxidant feature, is emerging as a promising approach for AMD treatment. Herein, a readily prepared antioxidant alginate-based hydrogel was developed to serve as a cytoprotective agent for RPE cells against oxidative damage. Alg-BOB was synthesized via conjugation of benzoxaborole (BOB) to the polysaccharide backbone. Hydrogels were formed through self-crosslinking of Alg-BOB based on benzoxaborole-diol complexation. The resulting hydrogel showed porous micro-structure, pH dependent mechanical strength and excellent self-healing, remolding, and injectable properties. Moreover, the hydrogel exhibited excellent cytocompatibility and could efficiently scavenge reactive oxygen species (ROS) to achieve an enhanced viability of ARPE-19 cells under oxidative condition. Altogether, our study reveals that the antioxidant Alg-BOB hydrogel represents an eligible candidate for RPE delivery and AMD treatment.

## 1. Introduction

Age-related macular degeneration (AMD) is one of the leading causes of blindness worldwide, affecting primarily people older than 55 years of age [1,2]. It has been estimated that by 2040, the total number of patients will increase to ~288 million [1]. AMD is featured by the accumulation of drusen between Bruch’s membrane and retinal pigment epithelium (RPE), which leads to progressive degeneration of photoreceptors and RPE, resulting in the loss of central vision [1,3]. RPE transplantation has been explored for the clinical treatment of AMD [4]. For example, human embryonic stem cells (hESCs) and induced pluripotent stem cells (iPSCs) have been reported to replace diseased RPE cells [5,6,7,8]. However, the treatment outcome by direct injection of RPE cell suspension is unsatisfactory due to drawbacks such as low cell viability, lack of suitable integration and cell distribution after the implantation to subretinal space [9]. The reconstruction of an appropriate extracellular matrix (ECM) is essential for the correct and organized development of functional cells in order to fulfill successful retinal tissue regeneration [6]. Various kinds of biomaterials and scaffolds, including natural polymers, synthetic polymers, decellularized tissues, and hydrogels, have been designed for retinal tissue engineering [5]. Among them, hydrogels are a kind of 3D crosslinked polymeric materials with ECM-mimicking mechanical property as well as microporous structure that allows for oxygen and nutrient permeation [10,11]. Hydrogels have been widely explored for drug delivery, 3D cell culture, and tissue engineering [12,13]. Recently, several studies have attempted to use hydrogels for RPE regeneration in vitro and in vivo [14,15,16,17,18]. For example, Molly et al. reported a hyaluronan-methylcellulose hydrogel for co-transplantation of both RPE and photoreceptors for vision repair [19].

It should be noted that native RPE cells are situated in a microenvironment with the highest oxygen tension of human body [14,20]. The retina has specific anatomical and metabolic characteristics including high oxygen consumption by the outer retina-RPE complex, high levels of cumulative irradiation, abundance of photosensitizers in the neurosensory retina and RPE, and so on [1]. Large amounts of reactive oxygen species (ROS) are produced during the physiological functions [3]. Moreover, RPE cells are sensitive to oxidative stress, and oxidative damage that leads to RPE dysfunction is believed to be strongly implicated in AMD pathogenesis [21,22,23,24]. Therefore, it is of great significance to rationally design hydrogels with ROS scavenging ability when used for RPE transplantation. However, only a few efforts were made on this issue, for example, hydrogels loaded with antioxidant cerium oxide nanoparticles were developed for RPE preservation against oxidative damage [25]. More attention should be paid to the exploration of novel antioxidant hydrogels for RPE cell cultivation and transplantation.

Phenylboronic ester (PBE), formed by complexation between phenylboronic acids (PBAs) and *cis*-diols, is one of the most common ROS responsive moieties [26,27]. Moreover, as a representative dynamic covalent bond, phenylboronic ester has been explored for preparation of hydrogels with excellent self-healing and injectable properties [28,29,30,31,32,33]. The formation of phenylboronic ester is pH-dependent and favours a solution pH higher than the p*K*_a_ value of PBAs [34]. However, a higher p*K*_a_ value (~8–9) above the physiological pH (7.4) is the “Achilles’ heel” for the majority of commonly used PBAs [35], impeding their potential application in biomedical areas. Benzoxaborole (BOB), an analogue of PBAs with a p*K*_a_ value of 7.2, has been developed by Hall et al. to show strong affinity to *cis*-diols at physiological pH [36]. Dynamic hydrogels based on benzoxaborolate crosslinking were further explored by Narain and others [37,38,39,40,41,42,43]. Similar to phenylboronic ester, the dynamic and reversible BOB-diol complexation also endowed hydrogels with excellent self-healing property [39], which is considered as an important element for advanced biomaterials. Moreover, recent studies have shown the ROS-responsiveness of benzoxaborolate crosslinked hydrogels [39,40]. Upon addition of H_2_O_2_, a typical kind of ROS, the hydrogels were gradually destroyed due to the degradation of BOB-diol complexation. Therefore, benzoxaborolate crosslinked hydrogels could serve as a potential candidate for RPE delivery with antioxidant feature.

Biocompatibility is a prerequisite for biomedical applications of hydrogels. Alginate, a natural water soluble polysaccharide with excellent biosafety and biodegradability, has been broadly developed as functional biomaterials for drug delivery, cell culture, and tissue engineering [44,45,46,47]. The abundant diol groups on the pyranose ring have been applied for formation of phenylboronic ester crosslinking to prepare hydrogels [34,48]. Moreover, alginate-based hydrogels have already been explored for RPE regeneration [15,16]. Nonetheless, hydrogels based on benzoxaborole-alginate diol crosslinking were not studied yet.

**Scheme 1:**
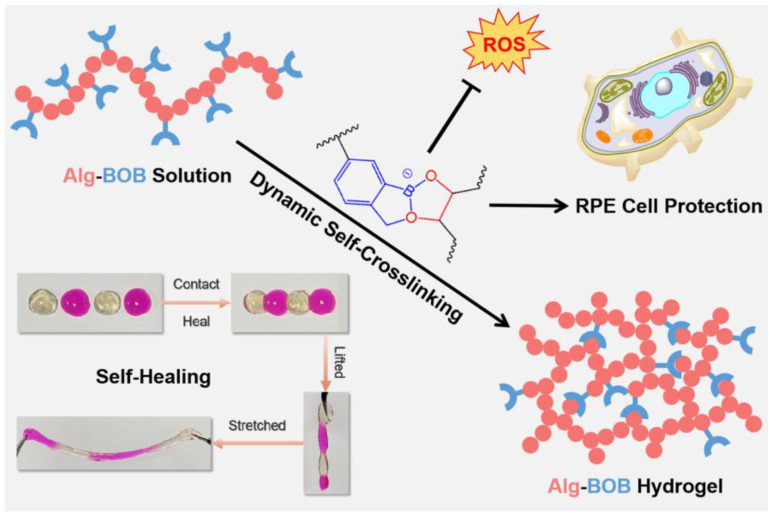
Schematic illustration of dynamically self-crosslinked Alg-BOB hydrogel with self-healing and antioxidant features for RPE cell protection against oxidative stress.

Herein, BOB conjugated alginate (Alg-BOB) was developed for facile preparation of hydrogels via dynamic BOB-diol complexation (Scheme 1). The pH regulated mechanical strength and self-healing ability were explored by rheological tests. The excellent reversible destroy-reconstruction behaviors such as self-repairing, remolding, and injectable properties, were further investigated by macro-observation. Moreover, 2,2′-azino-bis (3-ethylbenzothiazoline-6-sulfonic acid) (ABTS) decolorization assay was carried out to examine the radical scavenging capability of Alg-BOB. Additionally, the cytocompatibility of both Alg-BOB polymer and hydrogel extract was studied by using L929 and ARPE-19 cell lines. Finally, H_2_O_2_ stimulated ARPE-19 cells were used as an in vitro AMD model to investigate the ROS scavenging ability and cytoprotective effect of Alg-BOB hydrogel against oxidative damage.

## 2. Results and Discussion

### 2.1. Synthesis of Alg-BOB Polymer and Hydrogel

**Figure 1:**
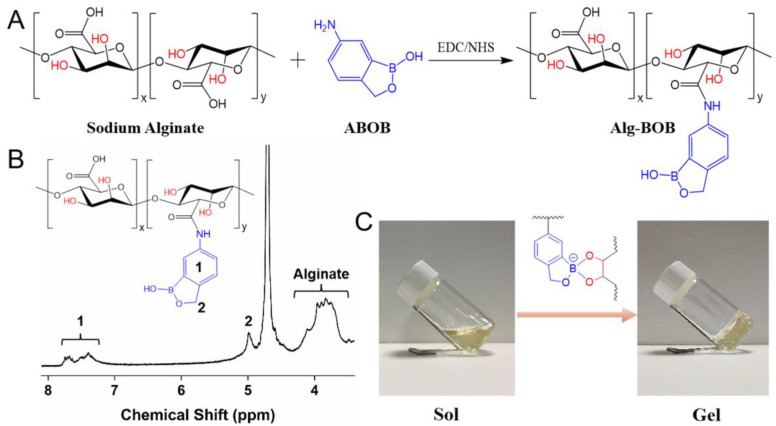
(**A**) Synthetic route of benzoxaborole conjugated alginate (Alg-BOB). (**B**) ^1^H NMR spectrum of Alg-BOB. (**C**) The sol-gel transition of Alg-BOB via dynamic crosslinking between BOB and alginate pyranose rings.

Alginate is a commonly used natural polysaccharide with excellent biocompatibility. The vicinal diols present in the repeating units of alginate backbone could be used for complexation with PBA to form reversible phenylboronic esters, while the carboxyl groups could be used for conjugation of bioactive molecules containing amino groups. In this study, BOB was grafted onto alginate by EDC/NHS chemistry activated amidation reaction (Figure 1A). The resulting Alg-BOB was obtained with a yield of around 83%. The chemical component of Alg-BOB was confirmed by ^1^H NMR. As shown in Figure 1B, typical proton peaks for both alginate pyranose ring (δ 3.56–4.25, 5H) and BOB (phenyl, δ 7.11–7.87, 3H; Ar–CH_2_–O, δ 4.99, 2H) could be clearly observed, demonstrating the successful conjugation of BOB. The peak position of BOB group here was in good agreement with that of BOB copolymers in our previous reports [37,49]. The degree of substitution (DS), i.e., the molar ratio of BOB unit to the carboxylate groups of alginate, was further determined to be ~22.4% by comparing the peak integral of BOB phenyl ring with that of alginate pyranose ring. The grafted BOB was believed to form dynamic complexation with the vicinal diol groups present on alginate pyranose rings, leading to the formation of polymeric networks.

Hydrogels based on crosslinking between BOB and diols, such as glycopolymer, catechol, and polyvinyl alcohol (PVA), have been reported by Chen and Narain et al. [37,38,39,50]. However, these hydrogels were all prepared by using at least two kinds of polymers. In comparison, Alg-BOB in this study was believed to form dynamic inter-/intra-molecular crosslinking between BOB and diols in the alginate backbone. This kind of self-crosslinking strategy offers a simpler methodology for hydrogel preparation. We found direct dissolution of Alg-BOB in pH 7.4 PBS did not lead to gelation because the presence of acidic alginate carboxyl groups declined the solution pH to be lower than p*K*_a_ value of BOB. Therefore, further addition of a few drops of 0.5 M NaOH basic solution was applied to increase pH and thus accelerate the formation of Alg-BOB hydrogel (Figure 1C). The gelation occurred rapidly (<30 s) via formation of dynamic benzoxaborolate interactions. For fabrication of hydrogels formed by conventional PBAs-diol complexations, NaOH basic solution was usually applied to tune the pH due to the high p*K*_a_ values (~8–9) of those PBAs. The resulting hydrogels were reported to exhibit good biocompatibility, even though these hydrogels were formed under weakly basic conditions [35,51,52]. In the present study, the lower p*K*_a_ value of BOB (~7.2) makes it possible to prepare hydrogels under physiological conditions by adding a limited amount of NaOH solution, revealing the advantage of BOB-diol crosslinked hydrogels for biomedical applications. Interestingly, we found that adding a high concentration of 10×PBS could also facilitate gel formation, probably due to intermolecular interactions between BOB and phosphate as enlightened by a previous report [53].

### 2.2. Characterization and Rheological Analysis of Alg-BOB Hydrogel

**Figure 2:**
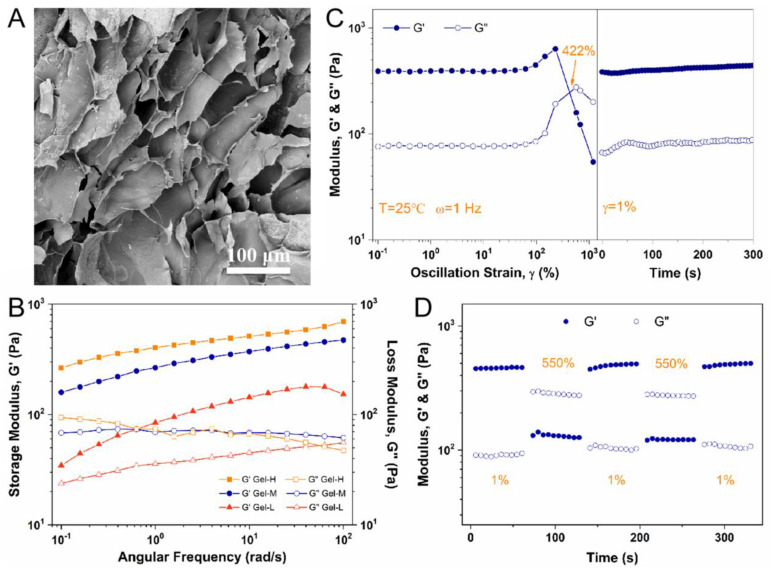
(**A**) Porous micro-structure of Alg-BOB hydrogel observed by SEM. (**B**) Mechanical strength of hydrogels prepared at different pH. (**C**) Oscillatory strain sweep from 0.1% to 1000% followed by an immediate time sweep for 300 s. (**D**) Step strain sweep at a low strain of 1% and a high strain of 550%.

The micro-structure of freeze-dried Alg-BOB hydrogel was observed by SEM. As shown in Figure 2A, the hydrogel displayed an interconnected porous network structure with an average pore size of 75.9 ± 25.7 µm, which is favorable for oxygen and nutrient exchange. The mechanical property of Alg-BOB hydrogel was studied by rheological tests. As is well known, the formation of BOB-diol complexation, similar to that of phenylboronic esters, is highly pH-dependent [34,40]. Therefore, the mechanical strength at different pH was compared by running frequency sweeps of Alg-BOB hydrogels. As shown in Figure 2B, the storage modulus for Gel-H, Gel-M, and Gel-L (ω = 10 rad/s, γ = 1%) was 511.8, 371.8, and 143.4 Pa, respectively. The higher modulus of hydrogel prepared by addition of more NaOH solution should be ascribed to the greater conversion to the benzoxaborolate complex at higher pH. The increased crosslinking density in the polymeric networks enhanced the hydrogel strength. This phenomenon was in good accordance with those benzoxaborolate crosslinked hydrogels in our previous reports [38,40,49]. Oscillatory strain sweep from 0.1% to 1000% at a fixed frequency of 1 Hz was also conducted (Figure 2C). Both G′ and G″ stayed steady at low strain (linear viscoelastic plateau) and then decreased dramatically along with an inversion of G″ exceeding G′, which indicated collapse of hydrogel network. The critical strain value for gel disruption (when G′ = G″) was confirmed to be approximately 422%. Interestingly, both G′ and G″ recovered almost 100% to the original values when a low strain of 1% was applied, suggesting a fast self-healing process of Alg-BOB hydrogel. Moreover, the fast reconstruction of hydrogel network (γ = 1%) after disruption (γ = 550%) was confirmed by step-strain sweep to be repeatable for at least 2 cyclic tests (Figure 2D). Therefore, this kind of fast and reproducible self-healing hydrogel based on self-crosslinking of Alg-BOB exhibits great potential for practical biomedical applications.

### 2.3. Self-Healing, Remolding, and Injectable Properties of Alg-BOB Hydrogel

**Figure 3:**
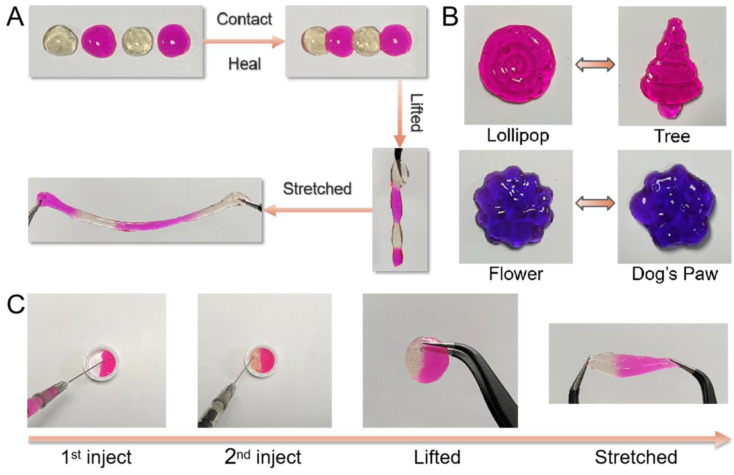
(**A**) Self-healing, (**B**) Remolding, and (**C**) Injectable properties of Alg-BOB hydrogel.

Hydrogels formed from dynamic covalent bonds are expected to have distinctive performances such as self-healing, remolding, and injectable properties. As confirmed by rheological study above, the fast formation–dissociation transition at molecular level of benzoxaborolate bonds led to efficient self-repairing of hydrogel. Here, macroscopic observation was further applied to provide a vivid demonstration of these unique features. As shown in Figure 3A, four pieces of hydrogels could be healed as one after contact for 1 min. The assembled hydrogel could even be lifted or stretched without any avulsion in the joint areas. The hydrogel could also be easily remolded into different shapes such as lollipop, tree, flower, and dog’s paw (Figure 3B). Moreover, the hydrogel could be injected via a 22G syringe, which is a typical feature for dynamic hydrogels classified as pseudoplastic (non-Newtonian fluid). Additionally, the broken hydrogel pieces could self-adaptively merged into one integral hydrogel disk with the same shape of the container (Figure 3C). The resultant hydrogel was strong enough to withstand a tensile force without disruption. These features should be attributed to the excellent reversibility of BOB-diol complexation in the hydrogel networks.

### 2.4. ROS Scavenging Ability

**Figure 4:**
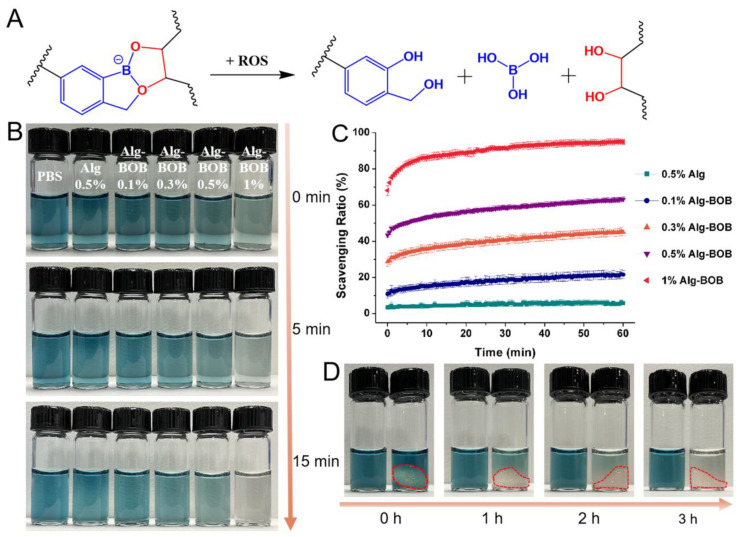
(**A**) Schematic illustration of the ROS responsive degradation of BOB-diol complexation. (**B**) ABTS^+•^ quenching ability of PBS, alginate, and Alg-BOB with different concentrations. (**C**) ABTS^+•^ scavenging ratio of alginate and Alg-BOB polymer solutions with different concentrations. (**D**) ABTS^+•^ radical scavenging ability of Alg-BOB hydrogel (marked by red dotted line). Left: PBS control, right: Alg-BOB hydrogel.

PBA and PBE have been widely used as ROS scavenger or for ROS responsive drug release [26]. BOB, a derivative of PBA, could be cleaved via oxidation to 2-hydroxybenzyl alcohol and boric acid as reported by Hall and co-workers [40]. The mechanism of ROS induced degradation of BOB-diol complexation is displayed in Figure 4A. Benzoxaborolate crosslinked hydrogels have been proved by our previous reports to show sensitivity to H_2_O_2_ [39,40], one of the most representative pathological ROS. Here, ABTS^+•^ was used to examine the radical scavenging ability of Alg-BOB polymer and hydrogel. As shown in Figure 4B, the color change of ABTS^+•^ showed a time and concentration dependent manner. The group with the highest concentration of 1% Alg-BOB showed the fastest clearance of ABTS^+•^ color within 15 min. However, negligible color change was found for PBS and alginate control groups, suggesting that the free radical scavenging performance should be attributed to the conjugated BOB moiety. The direct visual observation results were consistent with quantitative measurements of ABTS^+•^ scavenging ratio. As shown in Figure 4C, the ABTS^+•^ clearance speed and ratio were positively correlated with the concentration of Alg-BOB. The 1% Alg-BOB polymer solution rapidly scavenged lots of ABTS^+•^ in the first few minutes and the final clearance ratio at 1 h was calculated to be ~95%. In contrast, the pure 0.5% alginate solution without benzoxaborolate modification only had a limited ABTS^+•^ clearance ratio of ~6%. The ABTS^+•^ quenching ability for Alg-BOB hydrogel was also tested by incubating a hydrogel piece in ABTS^+•^ solution. Remarkable clearance of ABTS^+•^ color was observed after 3 h incubation (Figure 4D). The color change for hydrogel was slower than that for polymer solution, which could be explained by the slower penetration of ABTS^+•^ from outside to inside of hydrogel network. These results demonstrated that the self-crosslinked Alg-BOB hydrogel could serve as a sustained ROS scavenger for cell protection against microenvironmental oxidative stress.

### 2.5. In Vitro Biocompatibility

**Figure 5:**
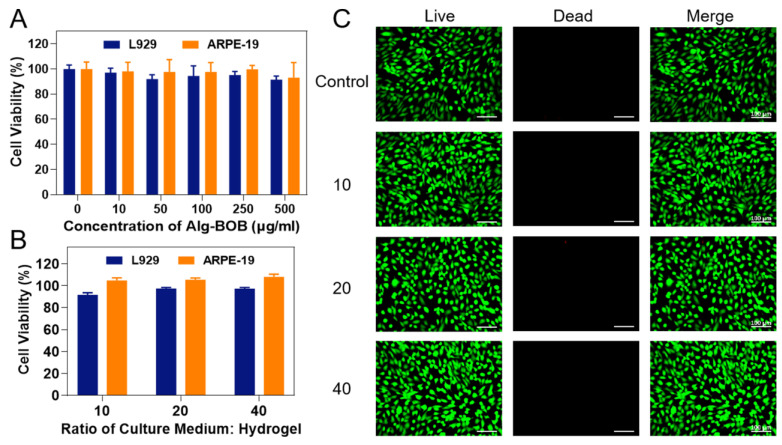
(**A**) Cell viability of L929 and ARPE-19 cell lines after co-incubation with Alg-BOB polymer solution for 48 h (*n* = 6). (**B**) Cell viability of L929 and ARPE-19 cell lines after co-culture with hydrogel extracts which were made by changing the ratio of culture medium: hydrogel (*n* = 5). (**C**) Live/Dead assay (green and red dots mean living and dead cells, respectively) of ARPE-19 cells cultured with gel extracts with different ratios of culture medium: hydrogel (10, 20, and 40, respectively). Scale bar = 100 μm.

For biomedical applications such as cell delivery, biocompatibility is an essential requirement to be considered. Cell Counting Kit-8 (CCK-8) assay and Live/Dead staining were performed to assess the cytocompatibility of Alg-BOB hydrogel. First, the cytotoxicity of Alg-BOB polymer was evaluated using both L929 and ARPE-19 cell lines (Figure 5A). After 48 h co-incubation, over 90% cell viability was achieved even at a concentration up to 500 μg/mL for both cell lines. Moreover, gel extracts prepared from different ratios of culture medium: hydrogel (10, 20, and 40, respectively) were used for cell culture for 48 h to evaluate the hydrogel cytocompatibility. As expected, gel extracts also exhibited negligible cytotoxicity to both cell lines (Figure 5B). In addition, the excellent biocompatibility of gel extracts was further confirmed by Live/Dead assay. As shown in Figure 5C, the vast majority of ARPE-19 cells were living cells and positively stained by calcein-AM (green fluorescence), whereas only very few dead cells were stained by EthD-1 (red fluorescence). Similar result was also found after co-incubation of L929 cells with gel extracts (Figure S2). These results were in accordance with previous reports that both alginate and BOB grafted polymers are bio-safe biomaterials for potential biomedical applications [41,44]. For further in vivo application, more detailed investigations including eye irritancy test and histopathological examination after intraocular administration should be conducted in our future study [54].

### 2.6. In Vitro ROS Scavenging and RPE Protection

**Figure 6:**
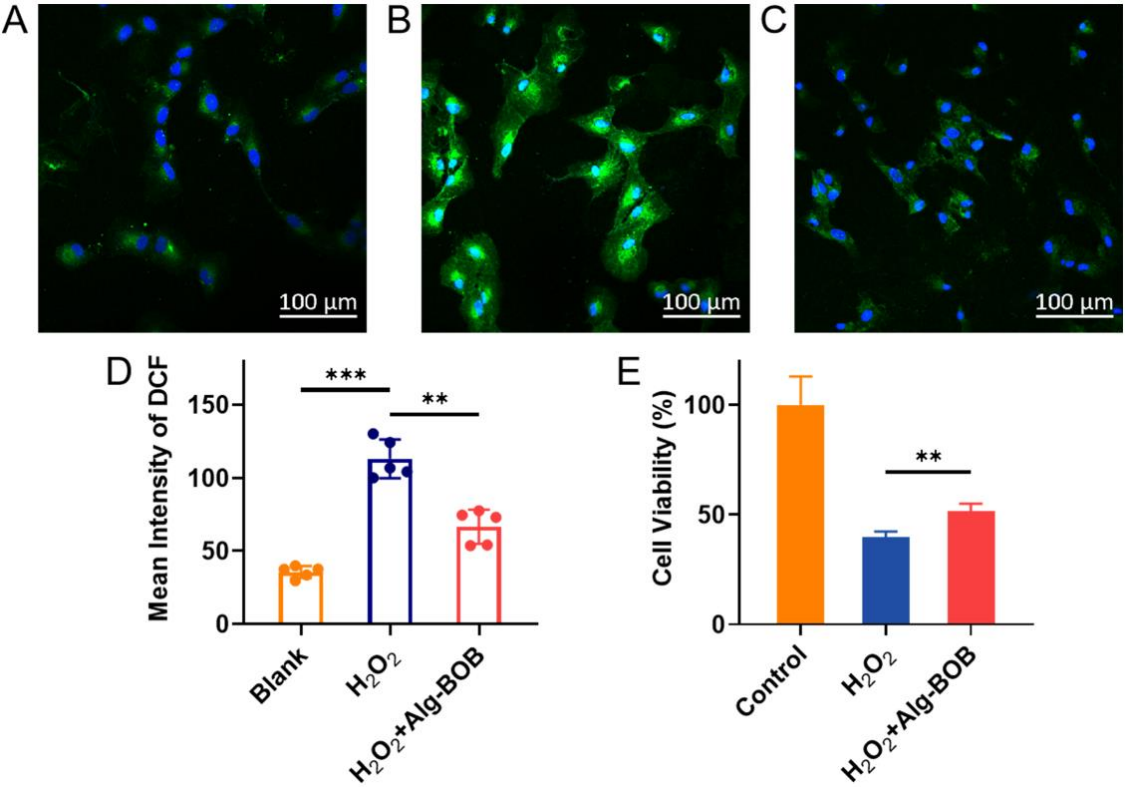
Fluorescence microscopy images showing the intracellular ROS levels of (**A**) Negative control with only culture medium, (**B**) Positive control (H_2_O_2_ stimulated for 4 h), and (**C**) Alg-BOB hydrogel co-incubated group with H_2_O_2_ treatment for 4 h. (**D**) Quantitative analysis of average DCF fluorescence intensity in each group (*n* = 5). (**E**) Cell viability of ARPE-19 cells post H_2_O_2_ treatment for 4 h with or without Alg-BOB hydrogel co-incubation (*n* = 5). Data represent mean ± SD, ** for *p* < 0.01 and *** for *p* < 0.005.

Encouraged by its satisfactory radical scavenging ability as mentioned above, Alg-BOB hydrogel may serve as a cell protecting agent against oxidative damage in vitro. Herein, H_2_O_2_ (400 μM) stimulated ARPE-19 cells were applied as an oxidative model to examine the ROS scavenging and cell protecting efficiency of Alg-BOB hydrogel. First, in vitro ROS scavenging ability was evaluated using 2′,7′-dichlorodihydrofluorescein diacetate (DCFH-DA) assay. As shown in Figure 6A–D, H_2_O_2_ treated cells exhibited a remarkable increase in 2′,7′-dichlorofluorescein (DCF) fluorescence intensity over cells treated with blank culture medium. By contrast, the addition of Alg-BOB hydrogel efficiently prevented the intracellular oxidative stress with a significant decline of DCF formation in cells. The cell protecting performance was further evaluated by using CCK-8 assay. As shown in Figure 6E, the cell viability of H_2_O_2_ treated ARPE-19 cells with or without addition of Alg-BOB hydrogel was 51.4% ± 3.7% and 39.7% ± 2.6%, respectively. The cell viability increased by around 29.5% benefiting from the addition of Alg-BOB hydrogel. The reduced intracellular DCF fluorescence intensity and enhanced cell viability should be attributed to the decreased extracellular H_2_O_2_ amount which was scavenged by Alg-BOB hydrogel in the transwell. Moreover, the gel extract made with H_2_O_2_ treatment (400 μM) was found to be nontoxic to both L929 and ARPE-19 cell lines (Figure S3), demonstrating the good bio-safety of degraded products of Alg-BOB. All these results suggested that Alg-BOB hydrogel could serve as an excellent antioxidant and cytoprotective agent for potential RPE cell delivery for AMD treatment.

## 3. Conclusions

In summary, we reported a facile method to prepare self-crosslinked alginate hydrogels based on dynamic BOB-diol complexation. The obtained Alg-BOB hydrogel exhibited excellent self-healing, remolding, and injectable properties due to the good reversibility of benzoxaborolate crosslinking, holding great potential for intravitreal injection in a minimally invasive way. The free radical scavenging ability of hydrogel, which is mainly related to the degradation of benzoxaborole groups, was confirmed by ABTS decolorization method. The 1% Alg-BOB polymer solution scavenged ~95% of ABTS radicals in 1 h. Alg-BOB hydrogel exhibited a slower ABTS radical clearance behaviour than its solution form, which could be beneficial for long-term sustained scavenging of microenvironmental oxidative stress in vivo. Moreover, intracellular ROS level was efficiently declined in 400 μM H_2_O_2_ stimulated ARPE-19 cells after co-incubation with Alg-BOB hydrogel, thereby preserving ~29.5% cell viability against oxidative injury. The reduced intracellular oxidative level and higher cell viability should be attributed to the decreased extracellular H_2_O_2_ amount with the aid of Alg-BOB hydrogel. Last but not least, both Alg-BOB polymer and Alg-BOB hydrogel extracts showed excellent cytocompatibility, even after 96 h long-term co-incubation. Therefore, our self-crosslinked antioxidant Alg-BOB hydrogel may serve as a promising candidate for treatment of AMD and other oxidative stress related retinal disorders.

## 4. Materials and Methods

### 4.1. Materials

6-Amino-1-hydroxy-2,1-benzoxaborolane hydrochloride (ABOB) was purchased from Bide Pharmatech Ltd. (Shanghai, China). Sodium alginate (200 ± 20 mPa·s), 1-(3-dimethylaminopropyl)-3-ethylcarbodiimide hydrochloride (EDC), and *N*-hydroxysuccinimide (NHS) were bought from Aladdin Reagents (Shanghai, China). Fetal bovine serum (FBS), penicillin−streptomycin, and DF12 cell culture medium were purchased from Gibco Life Technologies, Inc. (NY, USA). Other chemicals and reagents were of analytical grade, obtained commercially.

### 4.2. Fabrication and Characterization of Alg-BOB Hydrogel

#### 4.2.1. Synthesis and Characterization of Alg-BOB Polymer

Sodium alginate (1.00 g) was dissolved in 94 mL of deionized water (DI water) for 4 h. EDC (0.98 g) and NHS (0.30 g) dissolved in 6 mL of DI water were added into the solution to activate the carboxyl groups of alginate. After 1 h activation, ABOB (0.473 g) dissolved in a mixed solvent of DI water (5 mL) and *N,N*-dimethylformamide (DMF, 15 mL) was added to the mixture under stirring. The pH of the reaction mixture was monitored periodically with pH paper and maintained at 5-6 by adding 1 M NaOH or 1 M HCl solution. The reaction was kept for 24 h, followed by dialyzing against DI water for 5 days. Alg-BOB was obtained after lyophilization (~83% yield). The degree of substitution (DS) of BOB moiety was determined by ^1^H NMR (500 MHz, Bruker, Rheinstetten, Germany).

#### 4.2.2. Preparation of Alg-BOB Hydrogel

Typically, 20 mg of Alg-BOB was dissolved in 750 μL of DI water. Afterwards, 250 μL of 100 mM PBS (pH 7.4) was added to the above solution and Alg-BOB hydrogel (2%, *w*/*v*) was formed quickly within 30 s. After being stabilized for 30 min, manipulation of hydrogel such as elongation by tweezers, shape molding or injection through a 22G needle was performed.

For rheological analysis, hydrogels with different pH were further prepared by a simple mixing process of 1 mL of Alg-BOB (2%, *w*/*v*, in DI water) and different amount (20, 28, and 36 μL) of 0.5 M NaOH. The resultant hydrogels were denoted as Gel-L, Gel-M, and Gel-H, respectively.

#### 4.2.3. Morphology of Freeze-Dried Alg-BOB Hydrogel

For morphologic characterization, the Gel-M hydrogel was snap-frozen in liquid nitrogen. After freeze-drying, a small piece of the dried material was acquired manually and fixed on electro-conductive paste. After sputter-coating with gold, the microstructure of hydrogel was observed using a scanning electron microscope (SEM, PhenomScientific, Eindhoven, Netherlands).

#### 4.2.4. Rheological Studies

The mechanical and self-healing properties were studied on a rheometer (AR-2000, TA Instruments, DE, USA) equipped with a Peltier stage using a 25 mm parallel-plate configuration at 25 °C. To compare the mechanical properties, frequency sweeps of all the hydrogels were tested from 0.1 rad/s to 100 rad/s with a constant strain of 1%. Gel-M hydrogel was further used to study the self-healing ability. The hydrogel was firstly tested by oscillatory strain amplitude sweep from 0.1% to 1000% at a constant frequency of 1 Hz to determine the critical strain required for gel failure. Then, a step-strain test was performed by repeating large strain (550%, 60 s) for network disruption and small strain (1%, 60 s) for mechanical recovery.

#### 4.2.5. Injectable, Moldable, and Self-Healing Properties

To investigate the injectable and self-healing properties of Alg-BOB hydrogel, the general behavior of hydrogels after following steps were observed through a camera. For a better view of the gel junction, the hydrogels were either stained with Rhodamine B (RhB) or kept with their original color. Four separated hydrogels were connected with each other for 1 min, then were lifted and stretched horizontally by forceps to investigate the self-healing property. Next, to examine the moldable property, hydrogels (either stained with RhB or methylene blue) were placed in different containers and observed for their shape change. Furthermore, differently colored hydrogels were injected into round caps through a 22 G needle, then were lifted and stretched.

### 4.3. Antioxidant Property

ABTS decolorization assay were carried out on Alg-BOB hydrogel and Alg-BOB polymer solution. In order to determine the decolorization of ABTS^+•^, 60 µL of each following solutions dissolved in PBS: 1) Alginate (0.5%), 2) Alg-BOB solution at different concentration (1%, 0.5%, 0.3%, 0.1%), and 3) PBS mixed with 150 µL of ABTS^+•^ solution. The absorbance at 734 nm of ABTS^+•^ was measured by a SpectraMax M5 microplate reader (Molecular Device, CA, USA) at predetermined times in an hour.

### 4.4. In Vitro Cytocompatibility

#### 4.4.1. Cell Culture

Murine fibroblast cell line L929 was obtained from Procell Life Science and Technology Co., Ltd. (Wuhan, China). Human RPE cell line (ARPE-19) was obtained from ATCC (USA). Both cells were cultured in DMEM/F12 culture medium supplemented with 10% FBS and 1% penicillin-streptomycin (complete cell growth medium).

#### 4.4.2. Cell Cytotoxicity via Cell Counting Kit-8 (CCK-8)

The cell cytotoxicity of both Alg-BOB polymer and gel extract was determined by CCK-8 assay (Dojindo, Japan) according to manufacturer’s instruction. The gel extracts were prepared by immersing the hydrogel in different volumes of complete cell growth medium (1:10, 1:20, 1:40, *v*/*v*) for 24 h. L929 and RPE cells were seeded in 96-well plates (7000 cells per well) in 100 µL of culture medium and incubated for 24 h at 37 °C. Then, culture medium was replaced with 100 µL of fresh culture medium or gel extracts or culture medium containing different concentrations of hydrogel precursor polymers. The polymer solution and gel extract were sterilized via UV light irradiation in advance. For future in vivo study or potential translational research and commercialization, other sterilization methods such as ethylene oxide, gamma radiation and steam sterilization could also be investigated. At predetermined time points, cells were rinsed with PBS for 3 times, then added with 100 µL of test solution (CCK-8:DF12 = 1:10, *v*/*v*) and incubated for one more hour. The cell viability was determined by the ratio of O.D. values of cells treated with Alg-BOB polymer solution or gel extracts versus control group without treatment (both minus absorbance of test solution). The O.D. values were measured at 450 nm by a microplate reader.

#### 4.4.3. Live/Dead Staining

The in vitro cytocompatibility of gel extracts was further evaluated using Live/Dead Viability/Cytotoxicity Kit (Invitrogen). First, 2.5 µL of calcein AM (4 mM) and 10 µL of ethidium homodimer-1 (EthD-1, 2 mM) stock solutions were diluted into 5 mL of Dulbecco’s Phosphate Buffered Saline (DPBS) to obtain the working solution with approximately 2 μM calcein AM and 4 μM EthD-1. L929 and ARPE-19 cells were seeded and incubated as described above. After co-culture with gel extract for 48 h, cells were rinsed with PBS 3 times and incubated with 100 µL staining solution for 30 min. Images were taken by DMi8 (Leica, Wetzlar, Germany).

### 4.5. In Vitro ROS Scavenging and RPE Cell Protection by Hydrogel

In vitro antioxidant ability of Alg-BOB hydrogel was determined by Reactive Oxygen Species Assay Kit (Phygene Biotechnology Co, Ltd., Fujian, China). Intracellular ROS levels were investigated by measuring the oxidative conversion of 2′,7′-dichlorodihydrofluorescein diacetate (DCFH-DA) to green fluorescent 2′,7′-dichlorofluorescein (DCF). Specifically, RPE cells were seeded in cell slides in 24 well plate at a density of 30,000 cells per well. DCFH-DA was dissolved in DF12/DMEM to a final concentration of 10 μM. The cells were allowed to incubate with probe for 2 h, then washed three times with PBS and replaced with 1 mL of serum-free DF12 medium containing 400 μM H_2_O_2_. Meanwhile, one piece of transwell chamber containing 100 μL of 2% Alg-BOB hydrogel was carefully placed into each well. After 4 h, the medium was discarded, washed with PBS three times, and the antifluorescence quencher containing DAPI was used for mounting. The group treated with 400 μM of H_2_O_2_ in the absence of Alg-BOB hydrogel was set as a positive control and the group treated with only normal medium was set as a negative control. The intracellular fluorescence was excited at 488 nm and collected at 493–630 nm with 20× objective by a confocal microscope (LSM 880, ZEISS, Oberkochen, Germany). The results were analyzed by using ImageJ software. The results of mean intensity of DCF were expressed as the mean fluorescence intensity as arbitrary units per cell.

To further study the cell protection ability of Alg-BOB hdyrogel against ROS insult, RPE cells were cultured in 24-well plates as described above. After 24 h incubation, the culture medium was replaced with fresh medium containing 400 µM H_2_O_2_. Meanwhile, transwells filled with 100 μL of Alg-BOB hydrogel were added to each well. After 4 h co-culture, antioxidative protection effect was evaluated by measuring cell viability through CCK-8 assay. Cells cultured in complete medium were used as control. To study the cytotoxicity of degraded products of Alg-BOB hydrogel under oxidative condition, gel extracts were prepared with similar method to that in 2.4.2 but with 400 µM H_2_O_2_ treatment for 24 h. The cell viability of L929 and ARPE-19 cell lines after co-culture with the above gel extracts were measured by using CCK-8 assay after 24 h.

### 4.6. Statistical Analysis

All quantitative data were presented as the mean and standard deviation (SD) of results. GraphPad Prism (Version 8.3.0) was used for the statistical analysis. Significant differences between the groups were determined by one-way analysis of variance (ANOVA) at confidence levels of 95% (*, P < 0.05), 99% (**, P < 0.01) and 99.5% (***, P < 0.005).

## Data Availability

Not applicable.

## Supplementary Materials

The following supporting information can be downloaded at: https://www.mdpi.com/article/10.3390/gels9010024/s1, Figure S1. Cell viability of (A) ARPE-19 and (B) L929 cell lines after co-incubation with Alg-BOB polymer solution for a long time of 72 and 96 h, respectively (*n* = 6).; Figure S2. Live/Dead assay (green and red dots mean living and dead cells, respectively) of L929 cells cultured with gel extracts with different volume ratios of culture medium: hydrogel (10, 20, and 40, respectively). Scale bar = 100 μm; Figure S3. Cell viability of L929 and ARPE-19 cell lines after co-culture with gel extracts prepared by immersing the hydrogel in complete cell growth medium in presence of H_2_O_2_ for 24 h (*n* = 6).
