# Supplementary material for: Self-Healing Alginate Hydrogel Formed by Dynamic Benzoxaborolate Chemistry Protects Retinal Pigment Epithelium Cells against Oxidative Damage
Source: Gels. 2022 Dec 29;9(1):24. doi: 10.3390/gels9010024 (PMC9857501; doi:10.3390/gels9010024)
Supplement: Supplementary file 1 [file gels-09-00024-s001.zip › gels-2081670-supplementary.pdf]

gels

Article

Self-Healing Alginate Hydrogel Formed by Dynamic Benzoxaborolate Chemistry Protects Retinal Pigment Epithelium Cells against Oxidative Damage

Minhua Liu [1,†], Yate Huang [1,†], Chunwen Tao [1], Weijia Yang [1], Junrong Chen [1], Li Zhu [1], Tonghe Pan [1], Ravin Narain [4,*], Kaihui Nan [1,2,3,*] and Yangjun Chen [1,2,3,*]

1 State Key Laboratory of Ophthalmology, Optometry and Vision Science, School of Ophthalmology & Optometry, Wenzhou Medical University, Wenzhou, Zhejiang 325027, China
2 National Engineering Research Center of Ophthalmology and Optometry, School of Biomedical Engineering, Wenzhou Medical University, Wenzhou, Zhejiang 325027, China
3 National Clinical Research Center for Ocular Diseases, Affiliated Eye Hospital of Wenzhou Medical University, Wenzhou, Zhejiang 325027, China
4 Department of Chemical and Materials Engineering, University of Alberta, Edmonton, Alberta T6G 2G6, Canada
† These two authors contributed equally to this work.
* Correspondence: narain@ualberta.ca; nankh@163.com; chenyj@wmu.edu.cn

Supporting Information

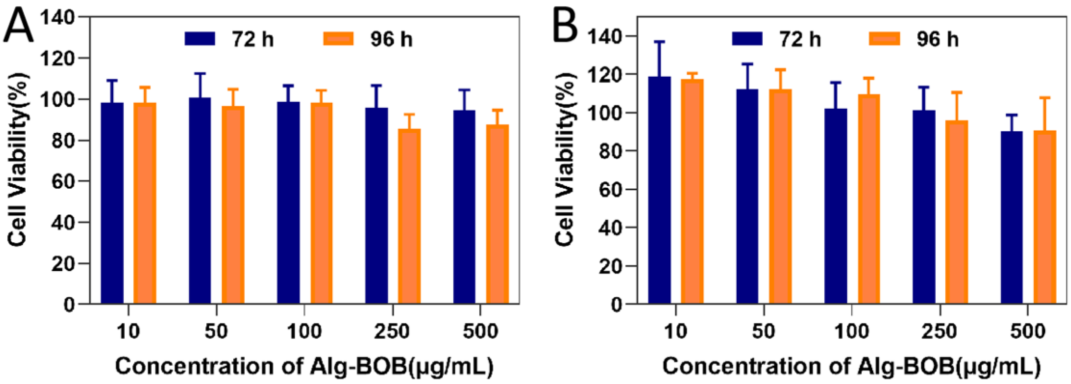

Figure S1. Cell viability of (**A**) ARPE-19 and (**B**) L929 cell lines after co-incubation with Alg-BOB polymer solution for a long time of 72 and 96 h, respectively ($n = 6$).

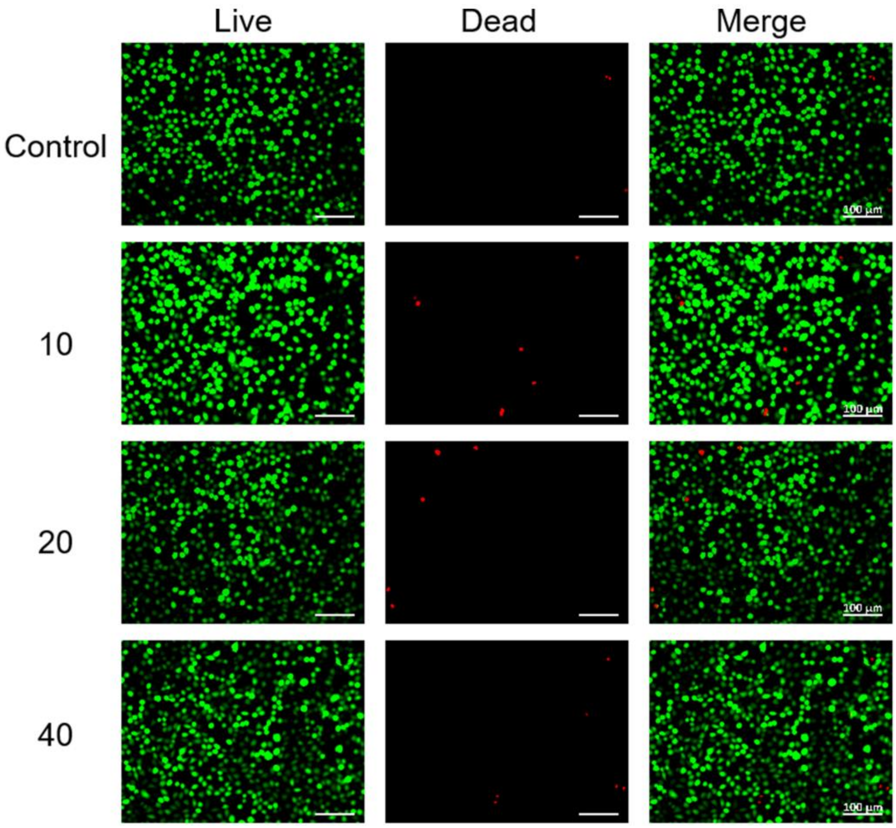

Figure S2. Live/Dead assay (green and red dots mean living and dead cells, respectively) of L929 cells cultured with gel extracts with different volume ratios of culture medium: hydrogel (10, 20, and 40, respectively). Scale bar = 100 μm.

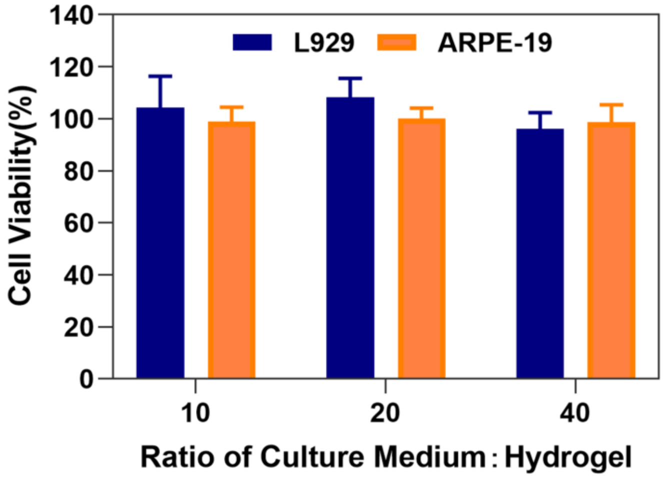

Figure S3. Cell viability of L929 and ARPE-19 cell lines after co-culture with gel extracts prepared by immersing the hydrogel in complete cell growth medium in presence of H_2O_2 for 24 h (n = 6).
